# Development of a Teen-Informed Coding Tool to Measure the Power of Food Advertisements

**DOI:** 10.3390/ijerph16214258

**Published:** 2019-11-02

**Authors:** Drew D. Bowman, Leia M. Minaker, Bonnie J. K. Simpson, Jason A. Gilliland

**Affiliations:** 1 Human Environments Analysis Laboratory, Western University, London, ON N6A 5C2, Canada; dbowman7@uwo.ca (D.D.B.); bonnie.simpson@uwo.ca (B.J.K.S.); lminaker@uwaterloo.ca (L.M.M.); 2 Children’s Health Research Institute, London, ON N6C 2V5, Canada; 3 Department of Geography, Western University, London, ON N6A 5C2, Canada; 4 School of Planning, Faculty of Environment, University of Waterloo, Waterloo, ON N2L 3G1, Canada; 5 DAN Department of Management and Organizational Studies, Western University, London, ON N6A 5C2, Canada; 6 School of Health Studies, Western University, London, ON N6A 3K7, Canada; 7 Department of Paediatrics, Western University, London, ON N6A 5C1, Canada; 8 Department of Epidemiology & Biostatistics, Western University, London, ON N6A 5C1, Canada

**Keywords:** food, food environment, information environment, advertising, marketing, food purchasing, adolescent, youth, public health

## Abstract

The food-related information environment, comprised of food and beverage advertising within one’s surroundings, is a growing concern for adolescent health given that food marketing disproportionately targets adolescents. Despite strong public interest concerning the effects of food marketing on child health, there is limited evidence focused on outdoor food advertising in relation to teenage diets, food purchasing, and perceptions. Further, limited research has considered both the exposure to and influence of such advertisements. This study used a novel multi-method approach to identify and quantify the features of outdoor food and beverage advertisements that are most effective at drawing teenagers into retail food establishments. An environmental audit of outdoor advertisements and consultations with youth were used to: (1) identify teen-directed food marketing techniques; (2) validate and weigh the power of individual advertising elements; and, (3) develop a teen-informed coding tool to measure the power of food-related advertisements. Results indicate that marketing power is a function of the presence and size of teen-directed advertisement features, and the relative nature of each feature is an important consideration. This study offers a quantitative measurement tool for food environment research and urges policymakers to consider teen-directed marketing when creating healthy communities.

## 1. Introduction

Poor nutrition contributes to several adverse health outcomes (e.g., obesity, cardiovascular disease, and type 2 diabetes) and is a leading cause of premature death [1,2,3,4]. The nutritional health of adolescents is a growing public health concern in many developed countries, as an estimated one-quarter to one-third of teenagers in high-income countries such as Australia, Canada, Ireland, New Zealand, the United Kingdom and the United States, are currently living with overweight or obesity [5,6,7]. An increasing body of literature recognizes the health impacts of food environments (FEs) [8,9,10], which are defined as the surroundings and conditions affecting one’s dietary patterns and nutritional health outcomes [11]. Studies have shown that youth’s FEs can be highly obesogenic, as they tend to lack easily accessible, nutritious food options, and expose teens to a wide selection of energy-dense, nutrient-poor foods [12,13]. This is especially concerning given that dietary habits formed early in life carry into adulthood [14,15,16]. 

Glanz and colleagues consider FEs, which they also refer to as “nutrition environments”, to contain a combination of four unique environmental elements: community (e.g., type and location of food vendors), consumer (e.g., pricing and availability of food items), organizational (e.g., school, work, home), and information (e.g., food advertising) [17]. While FEs are increasingly recognized as key determinants of community health [17,18], researchers have not yet considered the food-related *information environment* to the same extent that they have considered other elements of the FE [19]. Nevertheless, the information environment, which includes all food and beverage marketing and advertising within a community, is embedded within most daily spaces, such as those in and around schools, workplaces, and food stores [17,20]. Given the extent of the information environment, it is particularly important to understand its role in dietary health. 

While there are several factors within FEs that could influence nutrition [10,17], research indicates there has been an increase in the prevalence of calorie-dense, nutrient-poor food items being advertised to, and consumed by, young people [21,22,23,24,25,26]. Teenagers are particularly susceptible to food advertisements and marketing strategies, and there is evidence indicating that high school-aged adolescents spend most of their own money on low quality food and drink purchases [22,23,27]. It is suggested that teenagers are at a heightened risk of being influenced by marketing, as opposed to young children, who are still very much constrained by parental authority [10,28]. Studies have also demonstrated that children’s purchasing behaviours, perceptions, and consumption patterns are impacted by their FEs quite differently than teenagers [10,23,28,29]. Consequently, these two age groups should be studied separately [10]. Nevertheless, Elliot [30] highlights that many of the FE studies that include teenagers include children under 13 [10,29,31,32]. Despite the strong interest and emerging research concerning the effects of food marketing on child health, there is limited environmental and public health research that focuses on its potential impacts on teenage food purchasing perceptions and dietary behaviours [10,29,30,31,33,34]. 

When it comes to measuring the information environment within communities, there are two key components: the exposure to and power of advertisements [35,36,37,38]. The exposure to an advertisement can be quantified by its geographic “reach and frequency”, whereas the power is related to the “content, design, and execution” of the message [39] (p. 11). According to Prowse [37], the impact of food and beverage advertising techniques depends on these elements, or as the World Health Organization describes: “the media in which the communication message appears and its creative content” ([39], p. 8). However, given that FE research is still an emerging area, validated and standardized tools to measure and compare the four elements of FEs have not yet been developed [17,29,40,41,42]. Furthermore, the information environment is often excluded from past FE assessments and reviews in the geographic and public health literature [10,19,43]. 

Researchers have highlighted the scarce evidence involving adolescents’ exposure to outdoor food and beverage advertisements [19,44,45,46]. Many of the studies that do explore this exposure simply examine general geographic factors, like the pervasiveness and distribution of food advertisements within communities and across neighbourhood types [43,44,47,48,49]. While coding schemes to classify various child-directed advertising criteria have been developed [36,45,50,51], most studies simply coded for the presence or absence of each criterion [19,31,36,50,51]. Further, such coding schemes applied predetermined criteria instead of consulting target populations to determine what factors are important to them; this tailoring of criteria is important to fully understand how advertising influences specific populations of interest [23,30]. To date, researchers have not yet considered the relative influence of each criterion on teens’ perceived purchasing behaviours. This is an important gap, given that some features could be more conducive to teen food purchasing than others. 

There are several changes underway regarding food policy in Canada, with a focus on improving the FE and restricting food and beverage marketing to children under 13 years [35,52,53,54]. Advertising elements with child appeal have been identified by Health Canada as features such as images, colours, music, language, and the use of characters and premium offers [55]. While organizations have established the broad elements of “child-directed advertising”, there are no corresponding teen-directed marketing guidelines ([55], p. 7). Elliot [30,31] highlights the importance of understanding teen perspectives when it comes to food marketing perceptions, and states that this group should be consulted before marketing policy is developed. Other researchers support the studying of consumers’ perspectives [56], and advocate for qualitative marketing insight as to how and why consumers select certain foods [57].

There are calls from national and global organizations [38,55] for additional FE research that encompasses the information environment element, and to create validated tools that effectively quantify the exposure and power of food advertisements within different geographic settings [34,37,38]. There is also a clear need for the development and deployment of measurement tools that directly consider teen perspectives [34]. In an attempt to answer the calls of health organizations and fill existing research gaps, the purpose of this sequential mixed-method study was to use an evidence-based approach, incorporating an environmental audit and youth engagement, to develop and validate a tool for coding and quantifying the power of food and beverage advertisements in and around environments frequented by teens. The overarching aims of this research are twofold: (1) to understand how food and beverage advertising influences teens’ dietary purchasing perceptions, and (2) to create a validated, teen-informed coding tool to quantify the power of food advertisements and complement existing literature that focuses on exposure. 

## 2. Materials and Methods 

This paper uses a sequential mixed-method approach [58,59] to develop a food information environment measurement tool by: (1) conducting inventories of food advertisements through an environmental audit (quantitative); (2) developing initial tool criteria through a collaborative and participatory approach engaging teens from a Youth Advisory Council (qualitative); and (3) validating and weighing the power of individual elements of food advertisements using online surveys administered to a diverse group of teens (quantitative). The outcomes will be incorporated into a teen-informed coding tool for assessing the exposure and power of food and beverage advertising in the retail food environment. Ethics approval for this study was granted by Western University’s Non-Medical Research Ethics Board (NM-REB# 107034).

### 2.1. Food Information Environment Audit 

A food information environment audit was conducted within London (ON, Canada) between May and October 2018 to generate a comprehensive collection of photos of outdoor advertisements for food vendors, billboards, and transit shelters. A local food retailer database provided by the local public health unit was first used to identify food vendor locations within the study area. The vendor types included in the audit were: full-service restaurants, fast food restaurants, convenience stores, and grocery stores. These types were chosen based on consultations with local high school students, which included discussions regarding where they go to buy food and were analogous to those in previous studies [19,60]. Food vendor audits were undertaken within a socio-spatially stratified sample (low/middle/high income; urban/suburban) of six different areas covering 20% of the entire study area. A geographic information system (GIS) database provided by the Human Environments Analysis Laboratory was then used to locate and map 100% of billboard and transit shelters within the study area to capture additional sources of food and beverage advertising, and then a GIS-enabled smartphone application called ArcGIS Collector (ESRI, Redlands, CA, USA) was used by research assistants to collect the food advertising data from food vendors, billboards, and transit shelters in the field (i.e., on site). The purpose of this quantitative phase was to provide geographic exposure data involving the information environment, including a sample of outdoor food and beverage advertisements collected directly from the study area, to be used for additional qualitative exploration with local teenagers. The data collected in the audits will be used in this study to provide examples during the consultations with youth.

### 2.2. Consultations with Youth

A local Youth Advisory Council (YAC) was consulted to qualitatively examine the photos of food advertisements that were collected in the community via the food information environment audit. This council, made up of 14 high school students, represented a diversity of ages (13–19 years), genders (six boys and eight girls), and ethnicities. All members spoke English, but diversity of the council was also apparent through the other languages they speak at home, including Arabic, Bengali, French, German, Hindi, Korean, Malayalam, Punjabi, Spanish, Tagalog, Tamil, and Urdu. Although we do not know the parent/family incomes or other individual-level socioeconomic factors, members were selected from high schools from across the city (with urban, suburban, and rural catchment areas), representing a diversity of socioeconomic environments (low, middle, and high income neighbourhoods). 

#### 2.2.1. Participatory Method 

A collaborative participatory approach was used to uncover teen perceptions of food and beverage advertising influences to be able to conceptualize the power of marketing techniques [61,62]. The council worked with the primary author during multiple council meetings (held from October 2018 to March 2019) to develop a validated, teen-informed coding tool that could be used to measure the information environment. During the first few meetings, the youth were introduced to the tool-development project and discussions were held to better understand the information environment’s impact on teens. Through the collaboration process, they explored a variety of food and beverage advertisements that were collected in the audit to uncover teens’ perceptions of food advertising. The youth engaged in a variety of structured discussions and activities aimed at identifying which features of food advertisements are most important at capturing their attention and drawing them inside food vendors. The council members were provided with an initial list of existing coding criteria (e.g., health appeal, novelty, humour) derived from literature to review and discuss features perceived to be important from a teen perspective, and to establish criteria they felt were missing from previous coding techniques [36,39,55,63]. The qualitative discussions with the YAC were recorded with handwritten notes (i.e., meeting minutes). 

#### 2.2.2. Identifying Teen-Directed Coding Criteria 

For the purpose of this paper, teen-directed refers to features of food advertisements that the YAC consider to be targeted at teens and that influence them or their peers. To develop a teen-directed coding scheme, a photo analysis survey was first used to explore the features of food and beverage advertisements that draw teens into food stores, and better understand how local marketing exposure influences students’ food perceptions and their perceived level of engagement with local food vendors. The YAC completed this survey on tablets during one of their meetings. Members were given a random sample of ten food and beverage advertisement photos (out of a diverse sample of 25) to reflect on and assess the marketing techniques used to capture their attention and attract them inside food vendors (10 questions per advertisement). The advertisements used in the survey were selected from the audit described in Section 2.1. Each random sample included a variety of advertisement types (e.g., billboards, transit shelters, and vendor signage) and marketing techniques (e.g., celebrity tie-ins, deals, company logos). The survey was designed to have each photo assessed by at least four different YAC members. 

**Figure 1:**
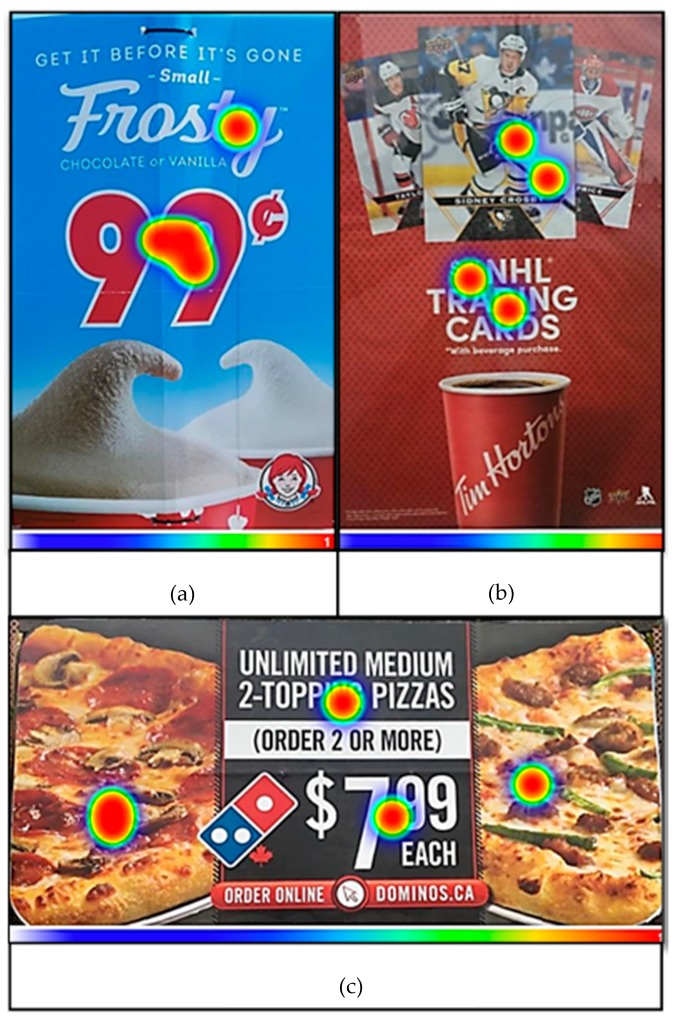
Teen-generated hot spots on vendor signage (**a**), transit shelters (**b**), and billboards (**c**).

A heat map technique was used to generate hotspots (depicted in Figure 1) which indicate the specific ad features that teens perceive to be the most important at capturing their attention. The teens generated these hotspots by clicking on the area of the advertisement that first captured their attention (i.e., What is the first thing that you see?). The colours in Figure 1 represent the frequency of the teens’ responses; the larger the red areas on the heat map, the greater number of respondents selected that specific area of the image. 

**Figure 2:**
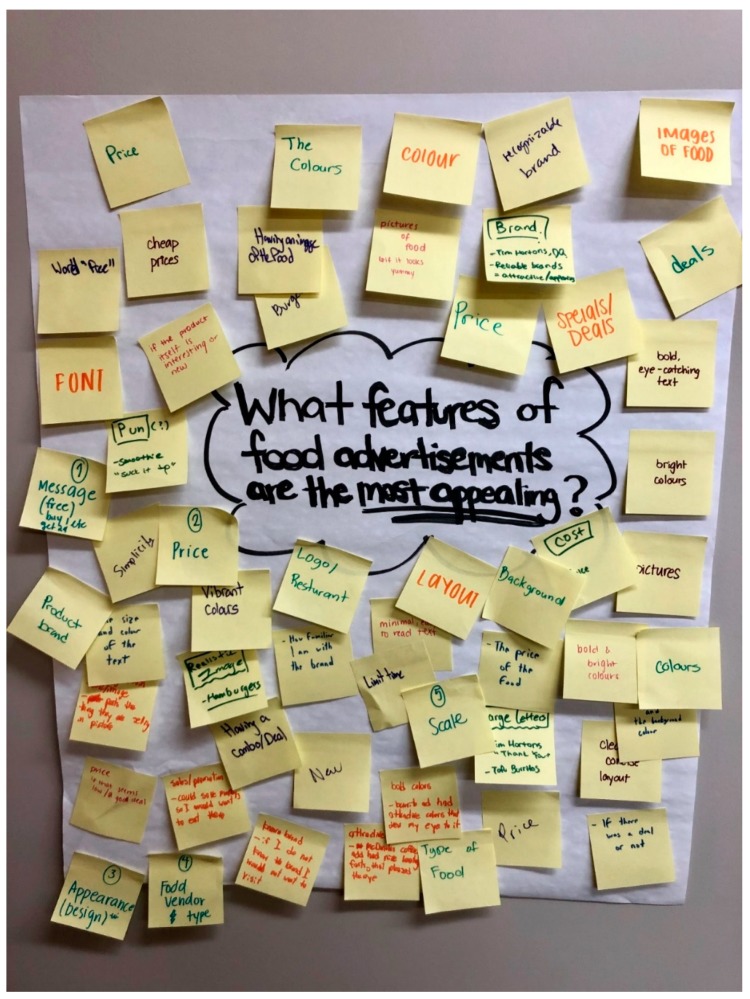
Youth advisory council members depict the top advertisement features that attract them and draw them inside food vendors.

Additional open-ended survey questions captured the parts of ads that (1) catch their attention the most, and (2) catch their attention the least. The teens were also asked to highlight the parts of the advertisements that are (1) appealing or (2) not appealing. Other questions used a 7-point Likert scale (i.e., 1 = “not at all” and 7 = “very much so”) to capture the perceived impact of viewing the advertisements, including perceived motivations to visit the featured store or buy a featured food item. During a council meeting, YAC members were also asked to independently write down the top five features of food and beverage advertisements that (1) attract them, and (2) draw them inside of food establishments, to further develop the tool’s coding criteria (See Figure 2). The top 10 teen-directed coding criteria that were identified by the local teens will be discussed in the results section, and these criteria were then used in the creation of the teen-developed weights discussed next.

### 2.3. Creating Teen-Developed Weights for Coding Tool Development

Upon consideration of the photo analysis survey results, coding literature, and structured discussions, the top ten criteria were identified by the YAC. After these criteria were identified, a second online weighting survey was developed and administered to a larger sample of Canadian teens (*n* = 44) to create teen-developed weights for each criterion. This sample included both the youth advisory council (*n* = 14) as well as participants who agreed to participate in focus groups for a larger project involving teens (*n* = 30). Other than age, no demographic information was collected for this phase of the study. This weighting survey was necessary, as it was evident from the YAC discussions that some criteria are generally more influential, or powerful, than others. 

The online weighting survey was designed to have participants weigh the ten criteria in three unique ways: (1) general ratings of importance, (2) relative rankings, and (3) point allocation. As Booysen has noted, “[a]nalysts today tend to experiment with a variety of weighting techniques and compare results across these techniques before selecting either one or a combination of techniques in deriving index estimates” ([64], p. 128). All three methods were used to assist with assigning weights to the coding tool, as described in the survey questions below.

(1) Assign each ad feature an individual rating out of 10 to describe its general level of importance. A score of 10 would indicate that the ad feature is extremely important.
(2) Rank the relative importance of each ad feature by arranging them in order from 1–10. 1 represents the MOST important ad feature and 10 represents the LEAST important ad feature.
(3) Distribute 100 points between the ad features, giving the most important feature(s) the greater number of points. When thinking about these ad features, please rate them according to their relative importance.

### 2.4. Analysis 

The qualitative discussions with the youth advisory council were recorded with handwritten notes and reviewed by the primary author. Member checking occurred at every stage of consultation to ensure that their views were accurately represented. The weighting survey data was exported into SPSS software for analysis. The relative weights for each teen-directed feature were captured in three different survey questions. When comparing the criteria, this notion of relativeness, or relative importance, refers to the idea that some criteria may be more important than others. Spearman’s correlation analysis was used to assess inter-rater reliability among the means. All three methods provided valuable information regarding these teen-directed coding criteria, but the point allocation captured specifically how much more or less important the features are in comparison to one another. This weighting method was ultimately used to develop the weights because it was an indication of relative importance; alternatively, the first survey question looked at general importance, and the second ranking question revealed the order of importance amongst the ten features. 

After the criteria were identified and the tool was developed, two research assistants were trained using the teens’ coding guidelines, and then they independently applied the coding tool to code every food and beverage advertisement captured in the environmental audits (*n* = 1092). After the coding was complete, a third coder finalized the codes and addressed all discrepancies that occurred between the initial coders. An inter-rater reliability (IRR) analysis was performed to evaluate the degree that the coders consistently measured the advertisement features (according to presence and size) using the developed tool. Kappa was calculated for each coder pair and the mean was used to produce a single index of IRR [65,66]. 

## 3. Results

### 3.1. Teen-Directed Marketing Criteria

Ten advertisement features identified by the youth advisory council as key teen-directed marketing techniques that attract teenagers to food establishments are shown below, in order of importance:

- Price
- Image of Food/Beverage
- Taste Description
- Sale/Deal/Special Offer
- Slogan/Description
- Logo/Company Name
- Geographic/Online Location or Directions
- Gamification (e.g., contest, game, giveaway)
- Loyalty Points/Rewards
- Character, Celebrity, or TV/Sports tie-in

Both the online survey results as well as the discussions with the youth revealed that these features are perceived by teenagers to be highly influential when it comes to perceptions of their own food purchases (see Table 1). For instance, prices are important to teens, who claim to be “more likely to go for stuff that’s cheaper”. Results also revealed that teens are often attracted to company logos; one teen mentioned that they “wouldn’t go if it didn’t look familiar”. Other elements from the literature that were initially considered by the youth council members include: (1) online/social media connections; (2) health appeal; (3) nutritional composition of items; (4) humour/clever language; (5) portrayal of values; (6) other enticements/amenities (e.g., wifi, parking); and (7) strange shapes or unusual colours. These criteria were discussed by the youth advisory council and were not deemed to be the most important influencers when it comes to their advertising and purchasing perceptions. 

It was found that certain criteria researchers consider to be teen-directed were not actually perceived to be important from a teen perspective. For instance, 13 of 14 youth advisory council members indicated that health appeal is not an important consideration in choosing where to eat out, and thus, it did not make the top ten criteria. Based on discussions with the youth advisory council, teens do not seem to be influenced by the healthfulness of the food items they purchase, as reflected in the comment, “if I’m going to eat out, I want to go all out”. Social media was another criterion that was not perceived by teens to be influential regarding their food purchases; several council members stated that they and their peers “do not use snapchap codes” or other methods of social media outreach. Although food vendors were the focus of this study, the discussions with teens also revealed that other community spaces where teens often congregate, including gaming centres and other settings that house teen-centred attractions, are common places where teens purchase food; for example, a council member said that their peers “like to eat at places like [Place X] because they have other attractions to do” (i.e., games, axe throwing, etc.). In a structured discussion, the youth advisory council determined definitions for the coding criteria, including inclusion/exclusion criteria and examples of these advertisement features. These criteria are outlined in Table 1 above.

Additionally, teens recognized that the appearance or layout of the ads (i.e., size, placement, and colour) plays a key role in the appeal of the food or beverage advertisement, with size being identified as the most important appearance-related feature. This became evident through the online photo analysis survey, with teens commonly indicating that the most attention-grabbing features of ads were often chosen because they are “the biggest part of the ad”, or because they “take up the most space” on the advertisement. Similarly, the least attention-grabbing features of ads were mainly selected due to their small size, often making them “too small in comparison to the rest” of the ad and “hard to read”. This reveals that the power of marketing techniques is not only a function of presence (whether each feature is present on the ad, and its relative importance), but also size. 

### 3.2. Teen-Developed Weights

Table 2 shows the teen-developed weights for each item, which represent the mean scores of each item based on the three weighting questions. The “order” refers to the order of importance, with 1 being the most important feature and 10 being the least important. Although point allocation was the weighting method chosen for this tool, teens rated the importance of the criteria similarly and consistently across all three survey questions (see Table 2). There was a high correlation between the three weighting methods (0.872–0.966). This adds to the rigor and reliability of the results and reveals that the three weighting methods are related.

**Figure 3:**
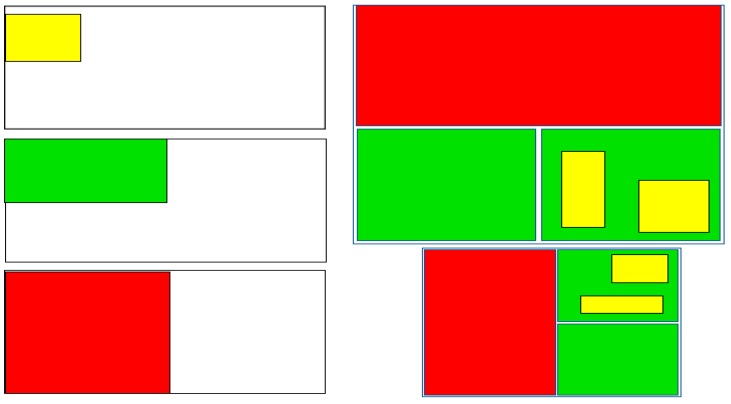
Sizing guide for the teen-informed coding tool. Note: There are three sizes: (1) Small, takes up less than ¼ of the ad (depicted in yellow), (2) Medium, takes up 1/4 of the ad (depicted in green), and (3) Large, takes up ½ of the ad or more (depicted in red).

The sizing diagram for the coding tool was originally developed by the research team (Figure 3). However, after additional consultations with teens regarding the size of ad features, it was decided the medium and large categories would be combined. The online hot spot activity particularly shed light on the importance of weighting on the basis of sizing, with teens rarely selecting the advertisement features that fell into the small categories as being attention-drawing. Thus, the following weights for sizing were created: small features get multiplied by 0.25, and anything larger (medium and large features) gets multiplied by 1.00. Absent features are coded as N/A (or 0).

### 3.3. Teen-Informed Coding Tool

Table 3 illustrates the mechanics of the coding tool, and how the power of each advertisement is generated. The power of an advertisement is derived from its overall score; for instance, a very powerful advertisement would have a high score (closer to 100) and a weak advertisement would have a low score (closer to 0). Based on the assigned weights for both the criteria and its sizing, this coding tool allows for the perfect score of 100 in the case that all 10 features are present and medium to large in size.

Results from the IRR analysis in Table 4 show that kappa was perfect (0.81–1) for all advertisement types, with kappa ranging from 0.893–0.910 [66], and where there was disagreement, a third coder from the authors acted as the decision maker. Percent agreement ranged from 96.2%–96.8%. This validates the reliability and replicability of the coding tool for measuring the power of several types of outdoor advertisements found within the information environment.

**Figure 4:**
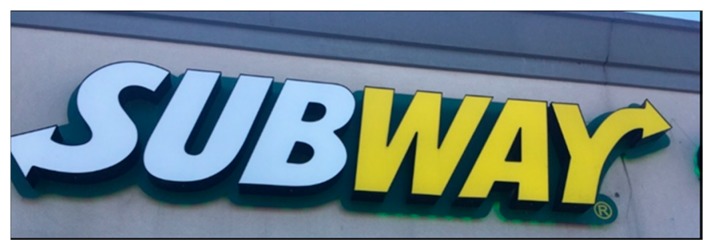
Advertisement A.

**Figure 5:**
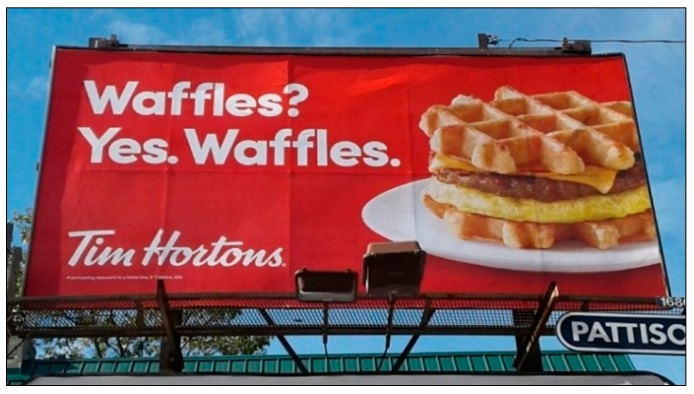
Advertisement B.

**Figure 6:**
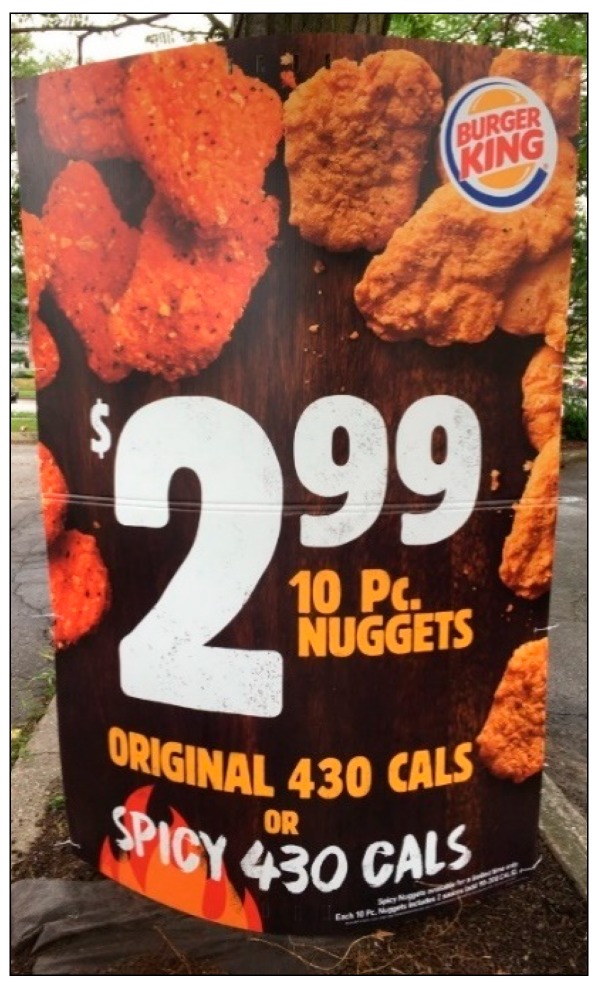
Advertisement C.

Although 100 is the highest score that is, in theory, achievable, it is unrealistic; when exploring and coding the advertisements with teens (*n* = 1092), this perfect score was never attained, and due to limited space on signage, it is unlikely that all ten features would be present as well as be considered ‘not small’. To demonstrate how this coding tool can be applied, a sample of three advertisements and the breakdown of their power scores is shown below (see Figure 4 with Table 5; Figure 5 with Table 6; and Figure 6 with Table 7). The ads range in power from low (6.93) to high (59.20).

## 4. Discussion

The current study investigated the food-related information environment in London, Ontario, Canada to create a validated, teen-informed coding tool that measures the power of food and beverage advertisements. The results pinpoint ten teen–directed criteria perceived to be most important in influencing teen purchases. Relativeness and size are also significant factors when it comes to teens’ perceived food purchases. For instance, the inclusion of both price and food image were much more important to teens than other advertisement features. Moving forward, this aspect of relative importance, as reflected in the tool’s weighting system, should be considered, since most studies solely code for the presence or absence of selected criteria without applying weights [36,50,51]. It was also recognized by the YAC, and consistent with the literature [36,37,38,39,67], that the size of ad features is an important consideration.

Our findings are consistent with the notion that children’s food purchasing perceptions are not necessarily influenced in the same way as teens’ [23,68]. For instance, toys and giveaways have been known to influence children’s perceptions and preferences [69,70,71], but gamification (defined in the context of this study as giveaways, contests, games) was shown to be of little importance to teens compared to many other advertisement features. In addition, our findings demonstrate that researchers’ perceptions of teen-directed advertising criteria do not always match up with teens’ perceived advertising influences. This was reflected in the youth advisory council discussions related to social media, when teens expressed that, although this feature is influential in connecting them with other people, it is not as important for teens connecting with food vendors or brands. Thus, this study reinforces the importance of conducting qualitative research with teenagers as a separate demographic [10,30].

The study makes contributions to both food environment and public health research and fills methodological and practical gaps within these fields. The findings address the limited research that measures the power of food and beverage related advertisements within different contexts [37] and how advertising affects teens’ purchasing perceptions [10,29,30,34]. The developed tool contributes to food environment methodology by providing a validated and objective means for quantifying the power of advertisements within and across neighbourhoods, from a teen perspective. Once applied, this coding tool produces an overall score that represents the power of each advertisement. Ultimately, the application of this tool can be used in research to demonstrate the pervasiveness of teen-directed food and beverage marketing and advocate for the restriction of marketing to teenagers. 

This contribution narrows the gap in food environment methodology, which currently lacks consistent and standardized measurement tools [17,29,40,41,42]. This tool will prove to be useful in geographic health research, as it allows for the combination of spatial mapping of advertising exposure through GIS technology, coupled with teenage perspectives to better understand the perceived impacts of marketing techniques; an incorporation of both qualitative and GIS methodology suggested by Riggsbee et al. [72] as necessary to achieve a more complete representation of young people’s food environments and their associated purchasing experiences and behaviours.

This research was needed to identify key teen-directed criteria to inform future food environment and marketing policy. For example, the results from this study can help inform federal Bill-S228, otherwise known as the Child Health Protection Act, aimed to prohibit food and beverage marketing to Canadian children under the age of 13 [53,73]. Currently, there are major concerns that this recent age amendment to Bill-S228, reduced from 17 to 13, will leave teenagers increasingly susceptible to teen-targeted marketing [30,52,54,74]. Thus, there is an increased focus on monitoring current advertising efforts to determine how advertising impacts teens in particular [52,74]. This controversy has led to large global and national organizations, including the World Health Organization [38,39] and Health Canada [35], issuing a call for additional research and evidence-informed guidance to support the development of an enabling food environment that promotes healthy diets and adequate nutrition. The United Nations Children’s Fund (UNICEF) [75] encourages the equal protection of all children, including teens, against marketing techniques, but expressed that despite this global push for the restriction of unhealthy food advertising to young people, it has not yet led to reduced exposure. Despite these shortcomings, this international organization continues to support policy limiting the exposure to, and power of, the information environment [75]. Thus, the findings from this study provide valuable insight on how to measure both the power of food advertisements and the extent of marketing exposure to Canadian teens, so that policymakers can understand how to reduce this type of exposure. 

This study has several limitations that must be considered. First, due to a small sample size, and a lack of demographic information beyond age, we do not have certainty that this tool is generalizable to the larger population of teens. The weights for size that are incorporated in this tool are not arbitrary, as they were decided on by a youth advisory council. However, considering teens often navigate their retail food environments from a distance (i.e., walking/biking from the sidewalk or street, driving by food vendor establishments and billboards) as opposed to viewing advertisements in close proximity, they often do not see the smaller features that could be seen by the youth advisory council in the research setting. This notion is reflected in many studies where they either exclude small advertisements from their design altogether, or code them according to size [46,48]. Research also shows that the perceptions of youth in London (ON, Canada) regarding safety and time constraints often limits active travel opportunities and results in increased driving [76]. Consequently, this justified our final weighting decisions for small, medium, and large features. Additionally, this study did not assign weights based on the advertisement type (i.e., billboard, vendor signage), but instead focused on exploring the content and size within each advertisement. Finally, this paper did not consider nutritional quality, considering it is already established that most food and beverages advertised to youth are low quality foods [22,24]. 

Future research should apply this teen-informed coding tool when conducting food information environment audits to quantify the power of food and beverage advertisements in diverse contexts and neighbourhoods surrounding teen-centered settings, such as high schools. The application of this tool in geographic research will provide important spatial and marketing information to federal health policymakers on the exposure and power of food-related marketing and may reveal its potential underestimation [37]. The use of this tool could also advocate for behavioural interventions and policy that reduce this type of exposure. Additional qualitative research is also needed to further explore teens’ perceptions and dive deeper into the teen-directed criteria that were identified. For instance, this research clearly demonstrates that price is an important feature to be present on an advertisement, but what is the price range that matters to teenagers and what is the cut off that would deter them from making a food purchase? Studies should further explore the relationship between advertisement power and spatial exposure in proximity to high schools. Construct validity needs to be examined regarding the relationship between teens’ exposures to different advertisements and their dietary intake. Ultimately, this research provides both methodological and practical contributions that will advance food environment research with a validated and objective measurement of the information environment.

## 5. Conclusions

This sequential mixed-method study incorporated a geographic food information environment audit and consultations with a local youth advisory council to explore how teens perceive the food information environment to influence their food purchasing behaviours. The youth advisory council explored a diverse sample of food and beverage related advertisement photos collected from London (ON, Canada), including billboards, transit shelters, and vendor signage. The coding tool that was developed in this study includes ten marketing criteria perceived to be the most important contributors to teens’ food purchase behaviours, and their relative weights. The results show that the importance of these criteria is relative, with some features proving to be more influential than others; thus, this aspect of relative importance should be considered in future food environment projects. Lastly, the findings reveal that the mixing of geographic and qualitative research to understand teens’ perceptions can be valuable for the health and wellbeing of teens. Future researchers may apply this tool to more accurately represent the exposure and power of food and beverage advertisements within communities, and to ultimately support policies that promote healthier behaviours within daily food environments.

## Figures and Tables

**Table 1:** Teen-developed coding guidelines.

Ad Feature	Definitions, Examples, Inclusion/Exclusion Criteria
1. Price	The price is present if it is available for a food/beverage item(s). Any other prices indicated on an ad (e.g., non-food items), are excluded. For example, $1 trading cards with beverage purchase would not be coded as “Price”—see features 4 and 8.
2. Image of Food/Beverage	The food/beverage image is present if there is a photo of a food or beverage on the ad. This does not include foods present in the logo of a vendor or brand.
3. Taste Description	The taste description/sensory appeal is present on the ad if there is a word(s) describing the taste (e.g., fresh, tasty, yummy, flavour, crunchy, delicious, ooey, gooey, savory). Note: Only words pertaining to taste are included.
4. Sale/Deal/Special Offer	There is a sale, deal, or special offer present if there is either: (1) a deal/discount towards a food or beverage item(s); or, (2) a limited time/special offer (e.g., limited time offer, this food is back from October–November 11th, get this summer special, seasonal offer, 2/$5, buy one get one free, reduced price, etc.). Exclusive specials on certain days of the weekday are also included (e.g., Tuesday sub of the day is lower price). Note: this is only for food/beverage items (i.e., if you get a free dilly bar with next purchase, this qualifies). However, if there is a limited time offer for a contest/giveaway that is not food or drink-specific (e.g., McDonalds Monopoly), but it also has a limited time date for the contest itself and not a specific food item, this is excluded— see feature 8.
5. Slogan/Description	The slogan/description is present if there is either: (1) a general ad description; (2) slogan or catchy phrase; or (3) a food or beverage item description (e.g., my summer tastes, handmade fresh tastes better, crispy chicken and waffle fries—also see feature 3). Note: most ads will have this feature present.
6. Logo/Company Name	The logo/company name is present if either the logo, company name, or food brand is included on the ad (e.g., Metro, President’s Choice, Dairy Queen, Loblaw’s).
7. Geographic/Online Location	The location is present if there is either the store address, directions, or website for ordering from the vendor. This ad criterion is more relevant for coding billboards and transit shelters. When coding food vendor signage that is already located at/on the property of the food store, this feature will be coded as N/A (0), unless it specifically has other location addresses on it or directions to other addresses. Online addresses qualify for ordering food/drinks online, like a web address or ordering app for a vendor, not a link for contests/giveaways—see feature 8.
8. Gamification	Gamification is present on this ad if there is a contest/game, giveaway, chance to win something (e.g., National Hockey League (NHL) trading cards, chance to win a dirt bike or trip with the purchase of certain food/beverage items). This is also likely coded as a description—see feature 5.
9. Loyalty Points/Rewards Program	Loyalty points/rewards program is present only if the ad mentions collecting points towards free food or being a member of a loyalty program (e.g., Petro Points, Student Price Cards, Scene Points, President’s Choice Optimum Points, MyWay Rewards, Pita Points). This is also likely coded as a description—see feature 5.
10. Character, Celebrity, or TV/Sports tie-in	Characters, celebrities, or TV/sports tie-ins are present if any of the following are present. This includes both people (e.g., a teenager) and cartoon characters on logos, including non-human characters used to promote a brand or food vendor (e.g., man on the KFC logo, Little Caesars pizza character, the slush puppy dog). Other examples that would qualify include: a person in the ad, Michael Jordan eating a burger, a photo from the Jurassic World movie, a professional chef, etc.

**Table 2:** Weighting methods for the coding tool (*n* = 44).

Ad Feature	Point Allocation		Rank		General Rating		
	Mean	Order	Mean	Inverted Order	Order	Mean	Order
Price	29.11	1	1.8	8.20	1	7.59	2
Image of Food/Beverage	20.80	2	2.09	7.91	2	7.84	1
Taste Description	11.73	3	5.07	4.93	4	6.16	4
Sale/Deal/Special Offer	9.98	4	4.48	5.52	3	6.75	3
Slogan/Item Description	8.52	5	5.36	4.64	6	4.84	6
Logo/Company Name	6.93	6	5.25	4.75	5	5.43	5
Location/Directions	4.27	7	6.89	3.11	7	4.39	7
Gamification	3.84	8	7.59	2.41	9	4.32	8
Loyalty/Rewards	3.30	9	7.16	2.84	8	4.84	6
Character, Celebrity, or TV/Sports tie-in	1.52	10	9.32	0.68	10	2.80	9

**Table 3:** Teen-informed coding tool for quantifying the power of food advertisements.

Ad Feature	Presence Yes(1) No (0)	Size ☐ ☐ ☐ NA (0) S (0.25) M/L (1)	Weight Totals (Presence × Size)
Price	____ × 29.11 ×	**☐ ☐ ☐**	=
Image of Food/Beverage	____ × 20.80 ×	**☐ ☐ ☐**	=
Taste Description	____ × 11.73 ×	**☐ ☐ ☐**	=
Sale/Deal/Special offers	____ × 9.98 ×	**☐ ☐ ☐**	=
Slogan/Item Description	____ × 8.52 ×	**☐ ☐ ☐**	=
Logo/Company Name	____ × 6.93 ×	**☐ ☐ ☐**	=
Directions/Location	____ × 4.27 ×	**☐ ☐ ☐**	=
Gamification	____ × 3.84 ×	**☐ ☐ ☐**	=
Loyalty Points/Rewards	____ × 3.30 ×	**☐ ☐ ☐**	=
Character, Celebrity or TV/Sports tie-in	____ × 1.52 ×	**☐ ☐ ☐**	=
Ad Power			= score/100

**Table 4:** Testing the inter-rater reliability of advertisement coding using a novel tool.

Feature	Kappa	% Agreement
Ad Type: Billboards/Transit Shelters (*n* = 93 ads)		
Price	0.910	96.8
Food/Beverage Image	1.000	100.0
Slogan/Description	0.891	95.7
Logo/Company Name	1.000	100.0
Location/Directions	0.950	97.8
Sale/Deal/Special Offer	0.731	92.5
Loyalty/Rewards	1.000	100.0
Gamification	0.903	98.9
Taste Description	0.678	91.4
Characters, Celebrities, TV, or Sports tie ins	0.863	94.6
Average	0.893	96.8
Ad Type: Outdoor Vendor Signage (*n* = 999 ads)		
Price	0.923	96.6
Food/Beverage Image	0.936	96.4
Slogan/Description	0.732	82.6
Logo/Company Name	0.884	92.6
Location/Directions	0.923	99.2
Sale/Deal/Special Offer	0.969	98.7
Loyalty/Rewards	0.998	99.8
Gamification	0.984	99.9
Taste Description	0.769	96.7
Characters, Celebrities, TV or Sports tie ins	0.980	99.7
Average	0.910	96.2

Note: For all inter-rater comparisons, *p* < 0.001.

**Table 5:** Coding example using the teen-developed tool: Advertisement A.

Ad Feature	Presence Score	Size Score	Totals (Presence Score × Size Score)
1. Price	0	0	0
2. Image of Food/Beverage	0	0	0
3. Taste Description	0	0	0
4. Sale/Deal/Special offers	0	0	0
5. Slogan/Description	0	0	0
6. Logo/Company Name	6.93	1	6.93
7. Directions/Location	0	0	0
8. Gamification	0	0	0
9. Loyalty Points/Rewards	0	0	0
10. Character, Celebrity or TV/Sports tie-in	0	0	0
**Total Power**			**6.93**

**Table 6:** Coding example using the teen-developed tool: Advertisement B.

Ad Feature	Presence Score	Size Score	Totals (Presence Score × Size Score)
1. Price	0	0	0
2. Image of Food/Beverage	20.80	1	20.80
3. Taste Description	0	0	0
4. Sale/Deal/Special offers	0	0	0
5. Slogan/Description	8.52	1	8.52
6. Logo/Company Name	6.93	0.25	1.73
7. Directions/Location	0	0	0
8. Gamification	0	0	0
9. Loyalty Points/Rewards	0	0	0
10. Character, Celebrity or TV/Sports tie-in	0	0	0
**Total Power**			**31.05**

**Table 7:** Coding example using the teen-developed tool: Advertisement C.

Ad Feature	Presence Score	Size Score	Totals (Presence Score × Size Score)
1. Price	29.11	1	29.11
2. Image of Food/Beverage	20.80	1	20.80
3. Taste Description	11.73	0.25	2.93
4. Sale/Deal/Special offers	9.98	0.25	2.50
5. Slogan/Description	8.52	0.25	2.13
6. Logo/Company Name	6.93	0.25	1.73
7. Directions/Location	0	0	0
8. Gamification	0	0	0
9. Loyalty Points/Rewards	0	0	0
10. Character, Celebrity or TV/Sports tie-in	0	0	0
**Total Power**			**59.20**

## References

[1] Ball G.D.C., McCargar L.J. (2003). Childhood obesity in Canada: A review of prevalence estimates and risk factors for cardiovascular diseases and type 2 diabetes. Can. J. Appl. Physiol..

[2] Lamichhane A.P., Mayer-Davis E.J., Puett R., Bottai M., Porter D.E., Liese A.D. (2012). Associations of Built Food Environment with Dietary Intake among Youth with Diabetes. J. Nutr. Educ. Behav..

[3] Morenga L.A.T., Howatson A.J., Jones R.M., Mann J. (2014). Dietary sugars and cardiometabolic risk: Systematic review and meta-analyses of randomized controlled trials of the effects on blood pressure and lipids. Am. J. Clin. Nutr..

[4] Public Health Agency of Canada (2017). How Healthy are Canadians? A Trend Analysis of the Health of Canadians From a Healthy Living and Chronic Disease Perspective.

[5] Bancej C., Jayabalasingham B., Wall R.W., Rao D.P., Do M.T., de Groh M., Jayaraman C. (2015). Evidence brief—Trends and projections of obesity among Canadians. Chronic Dis. Inj. Can..

[6] Rao D.P., Kropac E., Do M.T., Roberts K.C., Jayaraman G.C. (2016). Childhood overweight and obesity trends in Canada. Health Promot. Chronic Dis. Prev. Can..

[7] NCD Risk Factor Collaboration (2017). Worldwide trends in body-mass index, underweight, overweight, and obesity from 1975 to 2016: A pooled analysis of 2416 population-based measurement studies in 128.9 million children, adolescents, and adults. Lancet.

[8] Barrett M., Crozier S., Lewis D., Godfrey K., Robinson S., Cooper C., Vogel C. (2017). Greater access to healthy food outlets in the home and school environment is associated with better dietary quality in young children. Pub. Health Nutr..

[9] Dubreck C.M., Sadler R.C., Arku G., Seabrook J., Gilliland J.A. (2019). A comparative analysis of the restaurant consumer food environment in Rochester (NY, USA) and London (ON, Canada): Assessing children’s menus by neighbourhood socio-economic characteristics. Pub. Health Nutr..

[10] Engler-Stringer R., Le H., Gerrard A., Muhajarine N. (2014). The community and consumer food environment and children’s diet: A systematic review. BMC Public Health.

[11] Vandevijvere S., Dominick C., Devi A., Swinburn B. (2015). The healthy food environment policy index: Findings of an expert panel in New Zealand. Bull. World Health Org..

[12] Minaker L.M., Raine K.D. (2013). The Food Environment in Canada: The Problem, Solutions, and The Battle Ahead. Can. J. Diabetes.

[13] Sadler R.C., Clark A., Wilk P., O’Connor C., Gilliland J.A. (2016). Using GPS and activity tracking to reveal the influence of adolescents’ food environment exposure on junk food purchasing. Can. J. Public Health.

[14] Craigie A.M., Lake A.A., Kelly S.A., Adamson A.J., Mathers J.C. (2011). Tracking of obesity-related behaviours from childhood to adulthood: A systematic review. Maturitas.

[15] McKeown A., Nelson R. (2018). Independent decision making of adolescents regarding food choice. Int. J. Consum. Stud..

[16] Singh A.S., Mulder C., Twisk J.W.R., van Mechelen W., Chinapaw M.J.M. (2008). Tracking of childhood overweight into adulthood: A systematic review of the literature. Obes. Rev..

[17] Glanz K., Sallis J.F., Saelens B.E., Frank L.D. (2005). Healthy Nutrition Environments: Concepts and Measures. Am. J. Health Promot..

[18] Kirk S.F., Penny T.L., McHugh T.L. (2010). Characterizing the obesogenic environment: The state of the evidence with directions for future research. Obes. Rev..

[19] Velazquez C.E., Daepp M.I.G., Black J.L. (2019). Assessing exposure to food and beverage advertisements surrounding schools in Vancouver, BC. Health Place.

[20] Glanz K. (2009). Measuring Food Environments: A Historical Perspective. Am. J. Prev. Med..

[21] Bugge A.B. (2016). Food advertising towards children and young people in Norway. Appetite.

[22] Harris J.L., Brownell K.D., Bargh J.A. (2009). The Food Marketing Defense Model: Integrating Psychological Research to Protect Youth and Inform Public Policy. Soc. Issues Policy Rev..

[23] McGinnis J.M., Gootman J., Kraak V.I., Institute of Medicine (IOM) (2006). National academy of sciences, committee on food marketing and the diets of children and youth. Food Marketing to Children and Youth: Threat or Opportunity?.

[24] Sadeghirad B., Duhaney T., Motaghipisheh S., Campbell N.R.C., Johnston B.C. (2016). Influence of unhealthy food and beverage marketing on children’s dietary intake and preference: A systematic review and meta-analysis of randomized trials. Obes. Rev..

[25] Thai C.L., Serrano K.J., Yaroch A.L., Nebeling L., Oh A. (2017). Perceptions of Food Advertising and Association with Consumption of Energy-Dense Nutrient-Poor Foods Among Adolescents in the United States: Results from a National Survey. J. Health Commun.

[26] Velazquez C.E., Black J., Billette J., Ahmadi N., Chapman G.E. (2015). A Comparison of Dietary Practices at or En Route to School between Elementary and Secondary School Students in Vancouver, Canada. J. Acad. Nutr. Diet..

[27] Story M., French S. (2004). Food Advertising and Marketing Directed at Children and Adolescents in the US. Int. J. Behav. Nutr. Phys. Act..

[28] Kraak V., Pelletier D.L. (1998). The influence of Commercialism on the Food Purchasing Behavior of Children and Teenage Youth. Fam. Econ. Nutr. Rev..

[29] Williams J., Scarborough P., Matthews A., Cowburn G., Foster C., Roberts N., Rayner M. (2014). A systematic review of the influence of the retail food environment around schools on obesity-related outcomes. Obes. Rev..

[30] Elliot C. (2016). Knowledge needs and the ‘savvy’ child: Teenager perspectives on banning food marketing to children. Crit. Public Health.

[31] Elliot C. (2014). Food as people: Teenagers’ perspectives on food personalities and implications for healthy eating. Soc. Sci. Med..

[32] Laska M.N., Hearst M.O., Forsyth A., Pasch K.E., Lytle L. (2010). Neighbourhood food environments: Are they associated with adolescent dietary intake, food purchases and weight status?. Public Health Nutr..

[33] Cawley J. (2006). Markets and Childhood Obesity Policy. Future Child..

[34] Velazquez C.E., Black J.I., Potvin Kent M. (2017). Food and Beverage Marketing in Schools: A Review of the Evidence. Int. J. Environ. Res. Public Health.

[35] Hooper M. (2018). Restricting the Marketing of Unhealthy Foods to Children in Canada: Update from Health Canada. https://interprofessional.ubc.ca/files/2018/11/Plenary_Hooper.pdf.

[36] Potvin Kent M., Martin C.L., Kent E.A. (2014). Changes in the volume, power and nutritional quality of foods marketed to children on television in Canada. Obesity.

[37] Prowse R. (2017). Food marketing to children in Canada: A settings-based scoping review on exposure, power and impact. Health Promot. Chronic Dis. Prev. Can..

[38] World Health Organization (WHO) (2019). Reducing the Impact of Marketing of Foods and Non-Alcoholic Beverages on Children.

[39] World Health Organization (WHO) (2012). A Framework for Implementing the Set of Recommendations on the Marketing of Foods and Non-Alcoholic Beverages to Children.

[40] Caspi C.E., Sorensen G., Subramanian S.V., Kawachi I. (2012). The local food environment and diet: A systematic review. Health Place.

[41] Kelly B., Flood V.M., Yeatman H. (2011). Measuring Local Food Environments: An overview of available methods and measures. Health Place.

[42] Lytle L.A., Sokol R.L. (2017). Measures of the food environment: A systematic review of the field, 2007–2015. Health Place.

[43] Minaker L., Shuh A., Olstad D., Engler-Stringer R., Black J., Mah C.L. (2016). Retail food environments research in Canada: A scoping review. Can. J. Public Health.

[44] Egli V., Zinn C., Mackay L., Donnellan N., Villanueva K., Mavoa S., Smith M. (2019). Viewing obesogenic advertising in children’s neighbourhoods using Google Street View. Geogr. Res..

[45] Herrera A.L., Pasch K.E. (2017). Targeting Hispanic adolescents with outdoor food & beverage advertising around schools. Ethn. Health.

[46] Yancey A.K., Cole B.J., Brown R., Williams J.D., Hillier A., Kline R.S., McCarthy W.J. (2009). A Cross-Sectional Prevalence Study of Ethnically Targeted and General Audience Outdoor Obesity-Related Advertising. Milbank Q..

[47] Cassady D.L., Liaw K., Miller S. (2015). Disparities in Obesity-Related Outdoor Advertising by Neighborhood Income and Race. J. Urban Health.

[48] Kelly B., Cretikos M., Rogers K., King L. (2008). The commercial food landscape: Outdoor food advertising around primary schools in Australia. Aust N. Z. J. Public Health.

[49] Parnell A., Edmunds M., Pierce H., Stoneham M. (2018). The volume and type of unhealthy bus shelter advertising around schools in Perth, Western Australia: Results from an explorative study. Health Promot. J. Austr..

[50] Elliot C. (2012). Packaging Fun: Analyzing Supermarket Food Messages Targeted at Children. Can. J. Commun..

[51] Potvin Kent M., Dubois L., Wanless A. (2012). A Nutritional Comparison of Foods and Beverages Marketed to Children in Two Advertising Policy Environments. Obesity.

[52] Health Canada (2019). Health Can Healthy Eat Strategy. https://www.canada.ca/en/services/health/campaigns/vision-healthy-canada/healthy-eating.html.

[53] Parliament of Canada (2019). Bill S-228: Act. Amend Food Drugs Act. Prohibiting Food Beverage Mark. Dir. Child. https://www.parl.ca/legisInfo/BillDetails.aspx?billId=8439397&Language=E.

[54] Potvin Kent M., Pauzé E. (2018). The Frequency and Healthfulness of Food and Beverages Advertised on Adolescents’ Preferred Web Sites in Canada. J. Adolesc. Health.

[55] Health Canada (2017). Consultation Report: Restricting Marketing of Unhealthy Food and Beverages to Children in Canada. https://www.canada.ca/en/health-canada/services/publications/food-nutrition/restricting-marketing-to-kids-what-we-heard.html.

[56] Bibeau W.S., Saksvig B.I., Gittelsohn J., Williams S., Jones L., Young D.R. (2012). Perceptions of the food marketing environment among African American teen girls and adults. Appetite.

[57] Lusk J.L., McCluskey J. (2018). Understanding the Impacts of Food Consumer Choice and Food Policy Outcomes. Appl. Econ. Perspect. Policy.

[58] Leech N.L., Onwuegbuzie A.J. (2009). A typology of mixed methods research designs. Qual Quant..

[59] Schoonenboom J., Johnson R.B. (2017). How to construct a mixed methods research design. Köln. Z. Soziol..

[60] Dubreck C.M., Sadler R.C., Arku G., Gilliland J.A. (2018). Examining community and consumer food environments for children: An urban-suburban-rural comparison in Southwestern Ontario. Soc. Sci. Med..

[61] Arunkumar K., Bowman D.D., Coen S.E., El-Bagdady M.A., Ergler C.R., Gilliland J.A., Mahmood A., Paul S. (2019). Conceptualizing Youth Participation in Children’s Health Research: Insights from a Youth-Driven Process for Developing a Youth Advisory Council. Children.

[62] Jagosh J., Macaulay A.C., Pluye P., Salsberg J., Bush P.L., Henderson J., Greenhalgh T. (2012). Uncovering the Benefits of Participatory Research: Implications of a Realist Review for Health Research and Practice. Milbank Q..

[63] Chang A., Schulz P.J., Schirato T., Hall B.J. (2018). Implicit Messages Regarding Unhealthy Foodstuffs in Chinese Television Advertisements: Increasing the Risk of Obesity. Int. J. Environ. Res. Public Health.

[64] Booysen F. (2002). An Overview and Evaluation of Composite Indices of Development. Soc. Indic. Res..

[65] Hallgren K.A. (2012). Computing Inter-Rater Reliability for Observational Data: An Overview and Tutorial. Tutor. Quant. Methods Psychol..

[66] Light R.J. (1971). Measures of response agreement for qualitative data: Some generalizations and alternatives. Psychol. Bull..

[67] Hutchings J.B. (1994). Food Colour And Appearance.

[68] Watts A.W., Miller J., Larson N.I., Eisenberg M.E., Story M.T., Neumark-Sztainer D. (2018). Multicontextual correlates of adolescent sugar-sweetened beverage intake. Eat. Behav..

[69] Boyland E.J., Halford J.C.G. (2013). Television advertising and branding. Effects on eating behaviour and food preferences in children. Appetite.

[70] Harris J.L., Pomeranz J.L., Lobstein T., Brownell K.D. (2009). A crisis in the marketplace: How food marketing contributes to childhood obesity and what can be done. Annu Rev. Public Health.

[71] Ohri-Vachaspati P., Isgor Z., Rimkus L., Powell L.M., Barker D.C., Chaloupka F.J. (2015). Child-Directed Marketing Inside and on the Exterior of Fast Food Restaurants. Am. J. Prev. Med..

[72] Riggsbee K.A., Riggsbee J., Vilaro M.J., Moret L., Spence M., Steeves E.A., Colby S. (2019). More than Fast Food: Development of a Story Map to Compare Adolescent Perceptions and Observations of Their Food Environments and Related Food Behaviors. Int. J. Environ. Res. Public.

[73] Vergeer L., Vanderlee L., Potvin Kent M., Mulligan C., L’Abbé M.R. (2018). The effectiveness of voluntary policies and commitments in restricting unhealthy food marketing to Canadian children on food company websites. Appl. Physiol. Nutr. Metab..

[74] Yan W., Hutchinson H. (2018). Health Canada Update: Healthy Eating Strategy. https://obrieniph.ucalgary.ca/files/iph/healthy-eating-strategy-and-planned-policy-hasan-hutchinson-and-willia.pdf.

[75] United Nations Children’s Fund (UNICEF) (2018). A Child Rights-Based Approach Food Mark: A Guide Policy Making.

[76] Wilson K., Clark A.F., Gilliland J.A. (2018). Understanding child and parent perceptions of barriers influencing children’s active school travel. BMC Public Health.

